# Challenges in Diagnosing Pleural Thickening: Primary Pleural Follicular Lymphoma

**DOI:** 10.7759/cureus.63018

**Published:** 2024-06-24

**Authors:** Daniil Katkov

**Affiliations:** 1 Department of Internal Medicine, Waterbury Hospital, Yale-Waterbury Internal Medicine Residency Program, Waterbury, USA

**Keywords:** pleural neoplasm, human immunodeficiency virus infection, pleural lymphoma, pleural thickening, primary follicular lymphoma

## Abstract

This is the case of a 66-year-old male with a medical history of HIV infection on combination antiretroviral therapy (cART) who presented to the hospital with gradually worsening chronic right-sided chest and abdominal pain over the past three months. Computed tomography (CT) with contrast showed new mass-like pleural thickening in the right lower lobe posteriorly with an associated small loculated right pleural effusion. A core needle pleural biopsy was performed, and the results were consistent with primary pleural malignant lymphoma. Histopathological and immunohistochemical examinations revealed CD10-positive, low-grade B-cell lymphoma. This case is considered a rare occurrence of primary malignant lymphoma developing in the pleura.

## Introduction

A rare case of non-pyothorax-associated B-cell non-Hodgkin’s pleural lymphoma in a patient with HIV is reported in this article. The diagnosis of pleural malignant lymphoma is often difficult due to its rarity and non-specific clinical presentation. The clinical manifestations can be vague, with patients potentially experiencing symptoms such as pleuritic chest pain, shortness of breath, cough, fever, anorexia, and weight loss [1-3].

Pleural disease associated with non-Hodgkin's lymphoma is well documented; however, solid pleural involvement is less common and usually occurs as a secondary condition. Primary pleural lymphomas are exceedingly rare [1-3]. Diffuse large B-cell lymphoma (DLBCL) represents the majority of primary pleural lymphoma cases, while follicular lymphoma is the second most common subtype [1].

Two main types of primary pleural lymphomas are described in the literature: body cavity-based lymphoma, often associated with HIV, and pyothorax-associated pleural lymphoma, linked to chronic inflammation from conditions such as tuberculosis. Body cavity-based lymphoma usually presents as effusions in the pleural, peritoneal, or pericardial cavities without a clearly detectable tumor mass and is often high-grade. Pyothorax-associated pleural lymphoma is related to chronic pleural inflammation and typically presents as a mass lesion [4].

## Case presentation

A 66-year-old male presented to the hospital with a three-month history of gradually worsening right-sided chest pain, right upper quadrant abdominal pain, episodic shortness of breath, an unproductive cough, and weight loss. His medical history was significant for HIV; he was diagnosed 34 years ago and managed with a combination of darunavir, cobicistat, emtricitabine, and tenofovir, with a recent undetectable viral load and decreased CD4+ count. He also had hepatitis C complicated by liver cirrhosis with esophageal varices, which was ultimately treated, opioid use disorder on methadone in remission, chronic obstructive pulmonary disease (COPD) not requiring home oxygen, nicotine dependence with a 120-pack-year history of smoking, and generalized anxiety disorder.

On admission, his vitals were unremarkable. Physical examination revealed some mild tenderness in the right upper quadrant with a negative Murphy’s sign, as well as some right-sided chest wall tenderness. His blood work was notable for mild normocytic anemia and mild thrombocytopenia. The complete metabolic panel was within normal limits.

**Figure 1:**
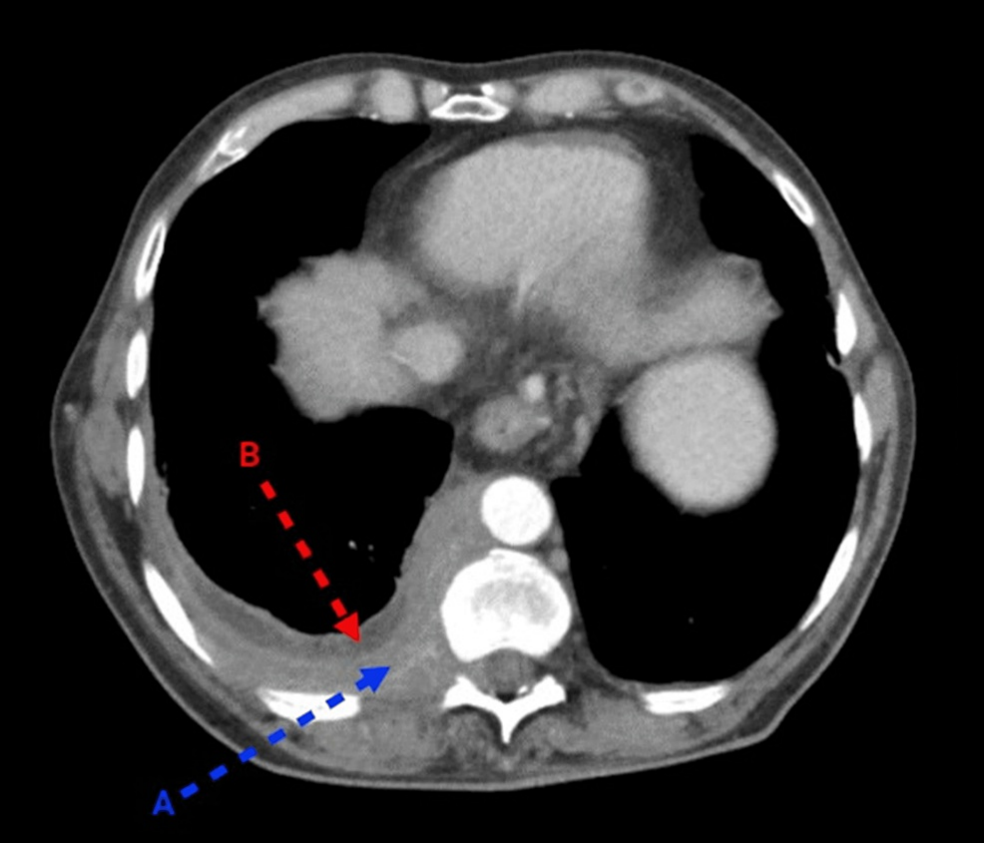
The patient's scan of the chest with intravenous contrast The blue arrow (A) points to pleural thickening; the red arrow (B) points to pleural effusion.

A chest radiograph showed a small, left-sided pleural effusion. An abdominal ultrasound was unremarkable. A computed tomography (CT) scan with contrast of the chest, abdomen, and pelvis revealed a small loculated right-sided pleural effusion and significant mass-like pleural thickening up to 2 cm along the right posterior lower chest (Figure 1). There were also findings consistent with emphysematous changes in both lungs, an enlarged spleen, liver cirrhosis, and prominent esophageal varices.

Diagnostic thoracentesis was performed, and pleural fluid analysis showed rare scattered lymphocytes, rare macrophages, and rare reactive-appearing mesothelial cells. Pleural fluid chemistry analysis showed elevated lactate dehydrogenase (LDH) levels and decreased glucose (Table 1).

**Table 1:** Pleural fluid analysis *Normal range is a protein content of less than 2% (1-2 g/dL); **Normal range for lactate dehydrogenase (LDH) in pleural fluid is considered to be less than 50% of the plasma level.

Laboratory parameter	Result	Normal range
Glucose (pleural fluid)	54 mg/dL	74-100
Total protein (pleural fluid)	2.0 g/dL	*
LDH (pleural fluid)	218 U/L	**
Total protein (serum)	7.1 g/dL	6.0-8.3
LDH (serum)	183 U/L	313-618
LDH (pleural fluid)/LDH (serum) ratio	1.19	<0.5
Total protein (pleural fluid)/ total protein (serum)	0.28	<0.5

**Figure 2:**
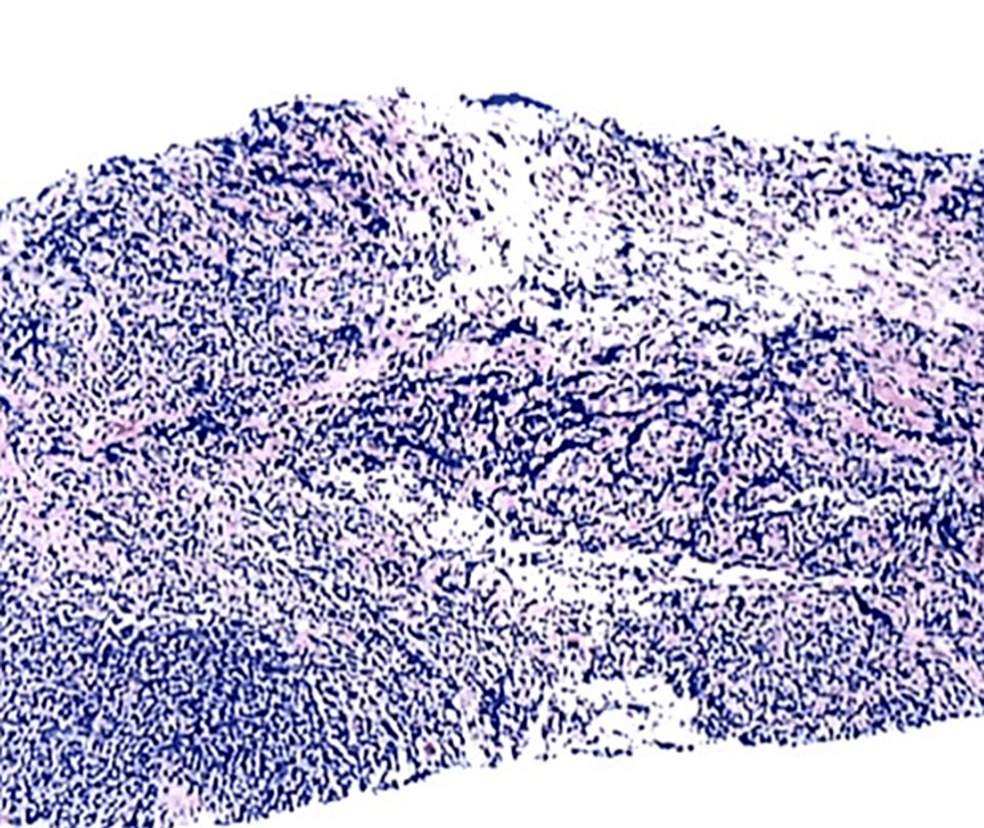
Pleural lymphoma (core biopsy of the presented patient); hematoxylin and eosin staining

A core needle biopsy of the pleural lesion was performed. Analysis of the sample showed cores of fibrous tissue infiltrated by an atypical lymphoid population comprised of small lymphocytes with scant to moderate cytoplasm and irregularly shaped nuclei with variable chromatin. Focal nodal formation was present, and no normal-appearing germinal centers were identified (Figure 2). By immunohistochemistry, the infiltrate was composed predominantly of B-cells with fewer T-cells (CD20 and CD3 immunostains, respectively). B-cells showed co-expression of CD10, BCL6, and BCL2. B-cells were also highlighted by PAX5 immunohistochemistry. The proliferation index was up to 70% by Ki-67 immunostaining. Immunohistochemistry for TTF-1, napsin-A, CD30, cyclin-D, and MUM-1 was negative. Immunohistochemistry for kappa and lambda chains demonstrated a mixed staining pattern with a slight kappa predominance. Fluorescence in situ hybridization (FISH) analysis revealed an IgH/BCL2 t(14;18) translocation. The diagnosis was most consistent with low-grade follicular lymphoma.

At the time of working on the case report, the patient refused any further diagnostic tests, including PET/CT, as well as any treatment for lymphoma.

## Discussion

Primary pleural lymphoma is an exceedingly rare condition, with the majority of cases typically involving secondary pleural involvement from systemic lymphomas. Primary pleural lymphomas account for only around 0.5% of all non-Hodgkin’s lymphoma cases and about 2.5% of primary chest wall tumors [1-3]. Diffuse large B-cell lymphomas constitute approximately 60% of primary pleural lymphoma cases. Follicular lymphoma represents the second most common subtype, accounting for about 20% of these cases [1].

Two main types of primary pleural lymphomas have been described in the literature: body cavity-based lymphoma, commonly associated with HIV, and pyothorax-associated pleural lymphoma, linked to chronic inflammation and, particularly, tuberculosis [4].

The first type, body cavity-based lymphoma, typically occurs in HIV patients and manifests as pleural, peritoneal, or pericardial effusions, usually without a clearly detectable tumor mass. This type of lymphoma is often high-grade and exhibits a B-cell or null-cell phenotype.

The second type is pyothorax-associated pleural lymphoma. The link between extranodal lymphomas and chronic inflammation is well documented, such as the connection between *Helicobacter pylori *infection and gastric lymphoma, as well as Hashimoto thyroiditis and primary thyroid lymphoma [5-7]. Similarly, this primary pleural lymphoma is linked to a history of chronic pyothorax or chronic pleural inflammation from tuberculosis [8]. Persistent pleural inflammation is believed to be a key factor in the development of these lymphomas. Pyothorax-associated pleural lymphomas usually present as a mass lesion and are high-grade non-Hodgkin’s lymphomas of B-cell origin [8].

In this case, a 66-year-old male presented with primary low-grade follicular pleural lymphoma. His long-standing HIV and extensive smoking history were likely significant factors in the pathogenesis. The histopathological findings, including the immunohistochemical profile showing CD20 positivity and co-expression of CD10, BCL6, and BCL2, alongside the presence of an IgH/BCL2 t(14;18) translocation, are characteristic of follicular lymphoma. This genetic translocation results in the overexpression of the BCL2 protein, which inhibits apoptosis, thus contributing to the prolonged survival of malignant B-cells [9].

## Conclusions

Here, a case of primary pleural low-grade follicular lymphoma was presented in a patient with long-standing HIV and a significant smoking history. This case underscores the rarity of primary pleural lymphoma and contributes to the limited body of literature on low-grade pleural lymphomas.

It highlights the importance of considering a broad differential diagnosis when evaluating pleural thickening and effusions in patients with complex medical histories. Further studies and case reports are needed to better understand the pathogenesis, clinical course, and optimal management strategies for this rare entity.
